# Gut-Protective and Multifunctional Exopolysaccharide from *Enterococcus faecium* HDRsEf1: Structural Characterization and Protective Effects Against Enteropathogenic *E. coli*-Induced Intestinal Inflammation

**DOI:** 10.3390/nu17233667

**Published:** 2025-11-24

**Authors:** Zeyuan Dong, Xinyang Li, Yaxin Wu, Zhaoyang Wang, Weitao Cui, Sishun Hu, Deshi Shi, Qi Huang, Yuncai Xiao, Hongbo Zhou, Zili Li, Zutao Zhou

**Affiliations:** 1 College of Veterinary Medicine, Huazhong Agricultural University, Wuhan 430070, China; dongzy@webmail.hzau.edu.cn (Z.D.); lxy3269735872@163.com (X.L.); 119623yx@webmail.hzau.edu.cn (Y.W.); 13615116152@163.com (Z.W.);; 2 Key Laboratory of Preventive Veterinary Medicine in Hubei Province, Huazhong Agricultural University, Wuhan 430070, China; 3 State Key Laboratory of Agricultural Microbiology, Huazhong Agricultural University, Wuhan 430070, China

**Keywords:** exopolysaccharides, *Enterococcus faecium*, enteropathogenic *Escherichia coli*, antibacterial activity, antibiofilm activity, antioxidant activity, intestinal health

## Abstract

Background: Enteropathogenic *Escherichia coli* (EPEC) disrupts intestinal barrier integrity by adhering to epithelial cells, leading to diarrhea, impaired nutrient absorption, oxidative stress, and intestinal inflammation in young animals. This study aimed to isolate and characterize a neutral exopolysaccharide (EPS-T1) from *Enterococcus faecium* HDRsEf1, evaluate its functional activities in vitro, and assess its protective effects against EPEC-induced enteritis in vivo. Results: EPS-T1, with a molecular weight of 81.21 ± 1.28 kDa, was mainly composed of glucose, galactose, rhamnose, and mannose, and exhibited a porous, sheet structure with relatively high thermal stability. In vitro, EPS-T1 (200 μg/mL) significantly inhibited EPEC growth and biofilm formation, reduced bacterial adhesion to intestinal epithelial cells, and exhibited broad-spectrum free radical scavenging activity. In vivo, EPS-T1 treatment alleviated EPEC-induced weight loss and intestinal tissue damage, reduced the intestinal EPEC load, downregulated pro-inflammatory cytokines (TNF-α, IL-1β, and IL-6), upregulated the anti-inflammatory cytokine IL-10, and improved serum antioxidant indices (T-AOC, SOD, and GSH-PX) while decreasing MDA levels. Conclusions: These results demonstrate that EPS-T1 derived from *Enterococcus* effectively mitigates EPEC-induced intestinal inflammation and oxidative stress, highlighting its potential as an immunobiotic functional candidate.

## 1. Introduction

The intestine plays a central role in maintaining host health by mediating nutrient absorption, immune regulation, and epithelial barrier integrity, thereby sustaining dynamic homeostasis with the external environment [1]. Disruption of these processes is closely associated with gastrointestinal infections, systemic inflammation, and metabolic diseases [2]. Among the various pathogenic factors, infection is a major cause of intestinal homeostasis disruption. Enteropathogenic *Escherichia coli* (EPEC) is a primary pathogen that causes infantile diarrhea and is highly virulent in neonates and young animals such as piglets and calves [3,4]. EPEC exerts its pathogenicity by adhering to intestinal epithelial cells and inducing attaching and effacing (A/E) lesions, thereby disrupting tight junction integrity and activating the NF-κB pathway, which leads to the overexpression of pro-inflammatory cytokines such as TNF-α and IL-6, exacerbating intestinal inflammation [5,6]. With the increasing prevalence of antibiotic resistance and the limited efficacy of traditional drug therapies, the development of safe and effective alternative prevention and treatment strategies has become an urgent research priority [7].

Therefore, bioactive exopolysaccharides (EPSs) derived from probiotics have attracted increasing attention as potential functional food ingredients for improving gut health and managing intestinal infections, owing to their excellent biocompatibility and multifunctional biological activities [8]. EPS produced by lactic acid bacteria (LAB) exhibit multiple biological functions, including broad-spectrum antibacterial activity [8,9], inhibition of pathogenic biofilm formation [10], antioxidant properties [11], and reduction in pathogen adhesion and anti-inflammatory properties [12]. Moreover, EPSs can function as prebiotics or postbiotics, modulating host immune responses and promoting gut microbiome homeostasis [13,14], making them promising natural alternatives to antibiotics and highlighting their application prospects in food safety and nutritional health.

Notably, the biological activity of EPS is highly dependent on their physicochemical properties and fine structure, including monosaccharide composition, molecular weight, glycosidic linkages, and polymer conformation [8,15]. Although extensive research has been conducted on LAB-derived EPS, most studies have focused on polysaccharides from the *Lactobacillus* spp. [16,17,18]. In contrast, systematic research on EPSs from *Enterococcus*, which are widely used in food fermentation and probiotic formulations, exhibits high environmental adaptability, remains poorly characterized [19] and is relatively limited. Importantly, few studies have systematically evaluated their functional effects in intestinal pathogen infection models via combined in vitro and in vivo approaches. This study aimed to systematically analyze the physicochemical properties of EPS produced by *E. faecium* HDRsEf1 and evaluate their protective effects on EPEC-induced intestinal injury. In vitro assays were conducted to assess EPS for antibacterial activity, inhibition of pathogenic biofilm formation, suppression of adhesion to intestinal epithelial cells, and antioxidant capacity. A mouse model of EPEC infection was subsequently used to evaluate the effects of EPS on pathogen colonization, inflammatory responses, oxidative stress, and intestinal tissue damage. By integrating structural characterization with in vitro and in vivo functional assessments, this research provides evidence for the structure–function relationships of *Enterococcus*-derived EPS and their potential applications in preventing intestinal infections.

## 2. Materials and Methods

### 2.1. Materials

Monosaccharide standards (L-arabinose, L-fucose, D-mannose, D-glucose, D-galactose, L-rhamnose, D-xylose, D-glucuronic acid, and D-galacturonic acid) were used. Reagents for free radical scavenging assays (catechol, ascorbic acid, hydrogen peroxide, ABTS, DPPH, and 1,10-phenanthroline) were purchased from Shanghai Macklin Biochemical Technology Co., Ltd. (Shanghai, China). Peptone and yeast extracts were obtained from Oxoid Ltd. (Basingstoke, Hampshire, UK), and brain heart infusion (BHI) broth was purchased from Qingdao Hope Bio-Technology Co., Ltd. (Qingdao, China). Unless otherwise specified, all other chemicals were of analytical grade or higher (Sinopharm Chemical Reagents Co., Ltd., Shanghai, China).

### 2.2. Bacterial Strains and Cells

*Enterococcus faecium* HDRsEf1, a patented strain in China, was isolated from the intestinal contents of local Chinese Tongcheng pigs [20]. EPS production was performed in modified MRS broth supplemented with 2% (*w*/*v*) glucose at 37 °C. The enteropathogenic *Escherichia coli* (EPEC66) strain (serotype O103:H4, sequence type ST642) was isolated from pork samples collected at a slaughterhouse in Yichang, Hubei Province, China [21]. For in vivo infection and real-time luminescence monitoring, a bioluminescent derivative of this strain, designated EPEC66-lux, was constructed via electroporation of a plasmid carrying the *luxCDABE* operon [21] and was subsequently grown in Luria–Bertani (LB) broth at 37 °C. Porcine ileal epithelial cells (IPI-2I) were maintained in DMEM containing 10% fetal bovine serum and antibiotics (100 U/mL penicillin and 100 μg/mL streptomycin) under 5% CO_2_ at 37 °C.

### 2.3. Extraction and Purification of Crude EPS

EPSs were extracted from *E. faecium* HDRsEf1 following the procedure of Kavitake et al. [15] with slight modifications. Briefly, logarithmic-phase bacteria of *E. faecium* HDRsEf1 were inoculated (2%, *v*/*v*) into modified MRS broth supplemented with 2% glucose and incubated at 37 °C for 24 h. After fermentation, the culture was centrifuged at 8000× *g* for 30 min at 4 °C to collect the supernatant, which was then heated at 100 °C for 10 min and cooled to room temperature. Proteins were removed by trichloroacetic acid (TCA) precipitation, and the resulting solution was subjected to ethanol precipitation and dialysis (molecular weight cutoff of 8000~14,000 Da, Solarbio, Beijing, China) to obtain crude EPS. The crude EPS was lyophilized and reconstituted into a 100 mg/mL solution and then filtered through a 0.22 μm membrane. For further purification, 10 mL of the filtrate was loaded onto a TOYOPEARL^®^ DEAE–650 M anion-exchange column (2.6 × 60 cm; Tosoh, Yamaguchi, Japan). Elution was performed stepwise with NaCl solutions of increasing concentrations (0–1.0 M) at a flow rate of 2.5 mL/min. At each salt concentration, 25 fractions (5 mL each) were collected. The carbohydrate content of each fraction was determined using the phenol–sulfuric acid method with glucose as a standard (Figure S1B, Supplementary Materials), and absorbance was measured at 490 nm. The major fraction showing the highest absorbance peak was collected, lyophilized, and designated as EPS-T1 and EPS-T2 for subsequent analyses.

### 2.4. Determination of Molecular Weight and Monosaccharide Composition

The molecular weights of EPS-T1 and EPS-T2 were determined by high-performance size-exclusion chromatography coupled with multiangle laser light scattering and refractive index detection (HPSEC-MALLS-RI) [22]. EPS-T1 and EPS-T2 were dissolved, filtered, and separated on SB-806HQ/SB-804HQ columns (Shodex, Tokyo, Japan), with detection via RI (Agilent, Beijing, China) and MALLS (Wyatt, Santa Barbara, CA, USA). The monosaccharide composition of EPS-T1 was analyzed via a Dionex ICS-6000 high-performance ion chromatography system (Thermo Fisher Scientific, Sunnyvale, CA, USA), with Rha, Ara, Xyl, Man, Gal, Gal, Fuc, GlcA, and GalA as standards for quantification [23,24]. The samples were hydrolyzed, dissolved in Milli-Q water, filtered, and injected for analysis.

### 2.5. Fourier-Transform Infrared Spectrometer (FT-IR) Analyses

Fourier transform infrared (FT-IR) spectroscopy was performed to analyze the functional groups of the purified EPS-T1 and EPS-T2 using a VERTEX 70 spectrometer (Bruker, Rheinstetten, Germany) [9]. The lyophilized sample was thoroughly mixed with spectroscopic-grade KBr and pressed into a transparent pellet approximately 1 mm thick. FT-IR spectra were recorded in the wavenumber range of 4000~400 cm^−1^.

### 2.6. Scanning Electron Microscopy (SEM)

The surface morphologies of EPS-T1 and EPS-T2 were observed via a scanning electron microscope (Quanta FEG 250, FEI, Hillsboro, OR, USA). A lyophilized sample was mounted on aluminum stubs with conductive carbon adhesive tape and sputter-coated with a thin gold layer to enhance conductivity before analysis. SEM images were captured at accelerating voltages of 30.0 kV.

### 2.7. Thermogravimetric (TG) and Differential Scanning Calorimetry (DSC) Analyses

The thermal properties of EPS-T1 and EPS-T2 were determined via thermogravimetric (TG) analysis, derivative thermogravimetry (DTG), and differential scanning calorimetry (DSC) via a thermogravimetric analyzer (STA 449 F3, NETZSCH Co., Ltd., Selb, Germany) [25]. The lyophilized sample was heated from 22 °C to 800 °C at 10 °C/min under a nitrogen atmosphere, and TG/DTG curves and DSC thermograms were recorded to assess the thermal stability and decomposition behavior.

### 2.8. Antibacterial Activity Assay

#### 2.8.1. Minimum Inhibitory Concentration (MIC) Determination

The minimum inhibitory concentration (MIC) of EPS-T1 against EPEC66-lux was determined via the broth microdilution method in 96-well microplates. EPS-T1 was serially diluted at 10 μg/mL intervals to obtain final concentrations ranging from 300 to 210 μg/mL. A bacterial suspension (~1 × 10^6^ CFU/mL) was added to each well to a final volume of 200 μL. Following incubation at 37 °C for 12 h, bacterial growth was evaluated on the basis of visual turbidity and optical density at 600 nm (OD_600_) via a Tecan Spark microplate reader (Tecan, Männedorf, Switzerland). The MIC was defined as the lowest EPS-T1 concentration at which no visible growth was observed, and the OD_600_ was significantly lower than that of the untreated control. Wells containing broth only or bacteria without EPS served as negative and positive controls, respectively.

#### 2.8.2. Oxford Cup Agar Diffusion Assay

The antibacterial activity of EPS-T1 was determined via the Oxford cup agar diffusion method. LB agar plates were uniformly inoculated with 100 µL of EPEC66-lux suspension (~1 × 10^6^ CFU/mL, logarithmic phase). Sterile Oxford cups were placed on the agar surface, and 200 µL of EPS-T1 solution at 50, 100, and 200 µg/mL was added and subsequently incubated at 37 °C for 24 h. Sterile distilled water served as the negative control. Zones of inhibition were measured to evaluate the antibacterial activity.

#### 2.8.3. Transmission Electron Microscopy (TEM)

EPEC66-lux was collected by centrifugation (5000× *g*, 5 min, 4 °C) and washed twice with phosphate-buffered saline (PBS, pH 7.4). A 200 µL aliquot of the bacterial suspension (~1 × 10^6^ CFU/mL) was incubated with EPS-T1 (100 and 200 µg/mL) at 37 °C for 12 h. Subsequently, the bacteria were fixed in 2.5% (*v*/*v*) glutaraldehyde in PBS at 4 °C for 2 h, washed, and placed onto carbon-coated copper grids. Negative staining was performed with 2% (*w*/*v*) phosphotungstic acid (pH 6.8) for 1 min. Excess stain was removed, and the samples were air-dried before examination under a transmission electron microscope (TEM, HT7800, Hitachi, Tokyo, Japan) operated at 80 kV.

### 2.9. Antibiofilm Activity Assay

The regulatory effects of EPS-T1 on EPEC66-lux biofilm development were evaluated via two methods: biofilm formation inhibition (coincubation) and mature biofilm disruption (post-attachment), following previously reported protocols [26]. For the biofilm formation assay, bacterial suspensions (1 mL, ~1 × 10^6^ CFU/mL) were co-incubated with EPS-T1 at final concentrations of 100 and 200 µg/mL in 12-well plates containing sterile glass coverslips and statically cultured at 37 °C for 48 h to allow biofilm development. For mature biofilm disruption, biofilms were initially formed under the same conditions for 48 h. The culture medium was then gently removed and replaced with medium containing EPS-T1 (100 and 200 µg/mL), followed by incubation at 37 °C for an additional 24 h. After treatment, the samples were gently washed with PBS to remove nonadherent cells. Biofilm bioluminescence was measured via an IVIS Spectrum imaging system (PerkinElmer, Waltham, MA, USA) to assess bacterial colonization. The biofilms were then fixed with methanol for 15 min, air-dried and stained with 0.1% (*w*/*v*) crystal violet for 15 min. After rinsing with PBS, biofilm morphology was observed under a light microscope. Finally, the bound crystal violet was solubilized with 33% glacial acetic acid, and the absorbance at 570 nm was measured to quantify the biofilm biomass. Parallel samples prepared under identical conditions were used for SEM. After being washed with PBS, the samples were fixed with 2.5% (*v*/*v*) glutaraldehyde in PBS at 4 °C. Fixed samples were dehydrated through a graded ethanol series (50% to 100%, 10 min per step), followed by critical point drying and gold sputter-coating. The biofilm ultrastructure was observed via a scanning electron microscope (SU3800, Hitachi, Tokyo, Japan).

### 2.10. Biosafety Evaluation

The biosafety of EPS-T1 was assessed via a previously published method with minor modifications [27]. Briefly, IPI-2I cells were seeded into 96-well plates and incubated overnight. The cells were then treated with increasing concentrations of EPS-T1 (0, 50, 100, 200, 250, 500, 750, 1000, and 2000 µg/mL) for 24 h. Gentamicin (100 µg/mL) served as a positive control. Subsequently, 10 µL of CCK-8 solution (Yeasen, Shanghai, China) was added to each well, and the plates were incubated at 37 °C in darkness for 2 h. The absorbance at 450 nm was measured via a Tecan Spark multimode microplate reader (Tecan, Männedorf, Switzerland).

### 2.11. Bacterial Adhesion Inhibition Assay

The effect of EPS-T1 on the adhesion of EPEC66-lux to IPI-2I cells was evaluated following previously described protocols with minor modifications [26,28]. Briefly, IPI-2I cells were seeded into 24-well black plates at a density of 2.5 × 10^5^ cells/well after trypsinization and cultured to approximately 85% confluence. Three treatment strategies were applied. In the competition assay, EPS-T1 (100 and 200 µg/mL) and EPEC66-lux (multiplicity of infection, MOI = 100) were simultaneously added and incubated for 2 h. In the exclusion assay, the cells were preincubated with EPS-T1 (100 and 200 µg/mL) for 2 h before inoculation, followed by an additional 2 h of coincubation. For the displacement assay, the cells were first exposed to EPEC66-lux for 2 h, after which the nonadherent bacteria were removed by washing with PBS, and EPS-T1 (100 and 200 µg/mL) was added for a further 2 h of incubation. All the treatments were incubated at 37 °C with 5% CO_2_. Following incubation, the samples were gently washed with PBS to remove nonadherent bacteria. Bacterial adhesion levels were assessed by measuring luminescence intensity via a Tecan Spark multimode microplate reader (Tecan, Männedorf, Switzerland) in luminescence mode (6000 ms/counts). Adherent bacteria were subsequently recovered by treating the cells with 0.1% trypsin, serially diluting them in PBS, and then plating them onto LB agar plates for counting colony-forming units (CFUs). Untreated cells served as the control.

### 2.12. Antioxidant Activity Assay

The antioxidant activity of EPS-T1 was evaluated via ABTS, DPPH, superoxide anion, and hydroxyl radical scavenging assays according to previously reported protocols with minor modifications [9,22,29].

#### 2.12.1. ABTS Radical Scavenging Activity

The ABTS solution (7 mM) was mixed with 2.45 mM K_2_S_2_O_8_ and incubated in the dark for 14 h. The mixture was diluted to an absorbance of 0.70 ± 0.05 at 734 nm. EPS-T1 solutions (250~2000 μg/mL, 100 μL) were mixed with 1.9 mL of the ABTS working solution for 6 min. The absorbance was measured at 734 nm (A_1_), and the scavenging activity was calculated as follows:

(1) $$\text{Scavenging}\;\text{activity}\;\left(\%\right)=\left(1\;-\frac{A_{1}-A_{2}}{A_{0}}\right)\;\times \;100\%$$

where A_1_ is the absorbance of the reaction mixture after treatment, A_0_ is the control absorbance, and A_2_ is the sample blank. Ascorbic acid was used as the positive control.

#### 2.12.2. DPPH Radical Scavenging Activity

EPS-T1 solutions (250~2000 μg/mL) were mixed with 0.2 mM DPPH methanolic solution (2:1) and incubated in the dark for 1 h. The absorbance was measured at 517 nm (A_1_), and the scavenging activity was calculated as described above.

#### 2.12.3. Superoxide Anion Radical Scavenging Activity

The reaction mixture contained 680 μL of Tris-HCl buffer (50 mM, pH 8.2), 100 μL of catechol (25 mM), and 200 μL of EPS-T1 (250~2000 μg/mL). After 4 min at room temperature, 20 μL of concentrated HCl was added to terminate the reaction. The absorbance was recorded at 320 nm (A_1_), and the scavenging activity was calculated as above.

#### 2.12.4. Hydroxyl Radical Scavenging Activity

The reaction mixture contained 200 μL of PBS (20 mM, pH 6.6), 100 μL of 1,10-phenanthroline (2.5 mM), 100 μL of FeSO_4_ (2.5 mM), 300 μL of H_2_O_2_ (0.1 mM), and 100 μL of EPS-T1 (250~2000 μg/mL). After incubation at 37 °C for 1 h in the dark, the absorbance was measured at 536 nm (A_1_). The hydroxyl radical scavenging activity (%) was calculated as follows:

(2) $$\text{Scavenging}\;\text{activity}\;\left(\%\right)=\left(\frac{A_{1}\;-{\;A}_{2}}{A_{0}-A_{2}}\right)\;\times \;100$$

### 2.13. Animal Experiments

#### 2.13.1. Animal Treatment and Administration

The mouse enteritis model was established via the luminescent *E. coli* EPEC66-lux strain following a previously described protocol with modifications [21,30], and preliminary studies confirmed that this strain exhibits moderate virulence; oral gavage with 10^9^ CFU induced soft stool formation in mice without causing mortality [21]. Therefore, 10^9^ CFU/mL was used as the infection dose in subsequent experiments. Four-week-old female ICR mice were housed under specific pathogen-free (SPF) conditions in barrier facilities at the Laboratory Animal Center of Huazhong Agricultural University, with free access to standard chow and water at a controlled temperature (22~25 °C) and a 12 h light/dark cycle. After 5 days of acclimatization, the mice were randomly assigned to five groups (*n* = 10 per group): the negative control group (NC), EPEC66-lux infection group (EPEP66), EPS treatment group (EP + EPS), probiotic treatment group (EP + EF1), and antibiotic treatment group (EP + AMC). All animal procedures were approved by the Institutional Animal Ethics Committee of Huazhong Agricultural University (Approval number: HZAUMO-2025-0110) and conducted under the Laboratory Animal Use License (SYXK [Hubei] 2020-0084) in accordance with the national guidelines for ethical review of animal welfare [31].

To induce infection, the mice were fasted for 12 h and gavaged with 0.2 mL of 3% NaHCO_3_ to neutralize the gastric acid. After 30 min, the mice in the treatment groups (except those in the NC group) were orally gavaged with 0.2 mL of EPEC66-lux suspension (~1 × 10^9^ CFU/mL in PBS) once daily for 3 consecutive days, whereas those in the NC group received sterile PBS. The following therapeutic interventions were administered for 5 consecutive days: EPS-T1 (200 mg/kg) for the EP + EPS group, EF1 suspension (1 × 10^9^ CFU/mL, 0.2 mL) for the EP + EF1 group and amoxicillin-clavulanate (40 mg/kg + 10 mg/kg) for the EP + AMC group. The experimental design is shown in Figure 8A. The body weights of the mice were recorded daily throughout the study, and their overall health status was monitored to assess potential adverse effects.

#### 2.13.2. In Vivo and Ex Vivo Imaging of the Intestine

The intestinal colonization of EPEC66-lux was monitored throughout the experimental period via the IVIS Spectrum imaging system (PerkinElmer, Waltham, MA, USA) to observe bacterial shedding and persistence. The mice were fasted for 12 h prior to imaging and anesthetized by inhalation of 2% isoflurane in oxygen. The abdominal bioluminescent signal was captured with a 1 min exposure time. The total photon flux (photons/s) in the abdominal region was quantified via Living Image software (version 4.4, PerkinElmer, Waltham, MA, USA) to evaluate bacterial colonization. For ex vivo imaging, the mice were first anesthetized with a mixture of 2% isoflurane in oxygen. Euthanasia was subsequently performed via the intraperitoneal injection of sodium pentobarbital (150 mg/kg). Death was confirmed by cessation of heartbeat and respiration, after which cervical dislocation was performed to ensure complete euthanasia. The entire intestine was then aseptically excised and placed in a sterile Petri dish for imaging under identical IVIS conditions.

#### 2.13.3. Fecal DNA Extraction and Quantification

The intestinal contents were collected, immediately snap-frozen in liquid nitrogen, and stored at −80 °C until further analysis. Total microbial DNA was isolated from the intestinal contents via the TIANNamp Stool DNA Kit (TIANGEN, Beijing, China) following the manufacturer’s protocol. DNA quality was assessed by measuring the A260/A280 ratios with a NanoDrop 2000 spectrophotometer (Thermo Fisher Scientific, Waltham, MA, USA). Quantitative PCR (qPCR) targeting the bacterial luciferase gene *luxA* was performed to quantify the EPEC66-lux bacterial load in the intestinal contents. The primers used were F: 5′-AAGTTGGCGTACTGGATGGG-3′ and R: 5′-AGGTCGCATCTCTGAGGAGT-3′. Each 20 μL reaction mixture contained 10 μL of 2× SYBR Green Pro Taq HS Premix (Accurate, Changsha, China), 0.4 μL of each primer (10 μM), 2 μL of the DNA template, and 7.2 μL of nuclease-free water. Thermal cycling was conducted on a Bio-Rad CFX Real-Time PCR system with initial denaturation at 95 °C for 5 min, followed by 40 cycles of 95 °C for 15 s and 60 °C for 30 s. Bacterial load was determined using a standard curve generated from serial dilutions of EPEC66-lux genomic DNA (Supplementary Materials Figure S2).

#### 2.13.4. Histological Examination

A portion of the intestinal tissue was fixed in 4% paraformaldehyde (PFA; Biosharp, Hefei, China) at room temperature. Fixed tissues were then embedded in paraffin, sectioned into thin slices, dewaxed in xylene, and stained with hematoxylin and eosin (H&E) for histopathological evaluation. Histopathological scoring of intestinal sections was performed on a 0–4 scale: 0 = intact villous epithelium, normal morphology; 1 = mild villus edema, epithelial collapse limited to the villus tips; 2 = mild necrosis of the mid-villus region; 3 = moderate necrosis of mid-villus region with visible crypt exposure; and 4 = severe villus necrosis with loss of epithelial structure. Two experienced researchers, blinded to the experimental groups, independently assessed the data [30,32]. The remaining intestinal samples were immediately snap-frozen in liquid nitrogen and stored at −80 °C for subsequent analysis.

#### 2.13.5. Cytokine Detection and Gene Expression Analysis

Frozen ileal tissues (200 mg) were minced, ground in liquid nitrogen, and homogenized in precooled PBS (0.01 M, pH 7.4) at 10% (*w*/*v*). The homogenates were centrifuged at 5000× *g* for 10 min at 4 °C, and the supernatants were collected for cytokine detection. The levels of TNF-α, IL-1β, IL-6, and IL-10 in the supernatants were determined via commercial ELISA kits according to the manufacturer’s instructions (Enova, Wuhan, China).

For RNA analysis, another 200 mg of frozen ileal tissue was processed via the RNAprep Pure Cell/Bacteria Kit (TIANGEN, Beijing, China) following the manufacturer’s protocol. The RNA concentration and purity were measured via a NanoDrop 2000 spectrophotometer (Thermo Fisher Scientific, Waltham, MA, USA). A total of 1.0 μg of RNA was treated with gDNA wiper to remove genomic DNA contamination, and cDNA was synthesized via the HiScript III 1st Strand cDNA Synthesis Kit (+ gDNA wiper) (Vazyme, Nanjing, China). The cDNA was stored at −20 °C until use. Quantitative real-time PCR was performed via the use of 2× SYBR Green Pro Taq HS Premix (Accurate, Changsha, China) on a CFX Real-Time PCR System (Bio-Rad, Hercules, CA, USA) under the conditions described in Section 2.13.3. The relative mRNA expression levels of TNF-α, IL-1β, IL-6 and IL-10 were calculated via the 2^−ΔΔCt^ method, with *Rplp0* used as the reference gene [33]. The primer sequences are listed in Table S1 (Supplementary Materials).

#### 2.13.6. Antioxidant Analysis

Blood was collected from the submandibular vein of anesthetized mice prior to euthanasia. The serum was separated by centrifugation at 3000 rpm for 10 min at 4 °C and immediately stored at −80 °C until analysis. The levels of total antioxidant capacity (T-AOC), superoxide dismutase (SOD), glutathione peroxidase (GSH-PX), and malondialdehyde (MDA) were quantified following the instructions of commercial reagent kits (Jiancheng, Nanjing, China).

### 2.14. Statistical Analysis

All in vitro data were represented and calculated from at least three replicates and are expressed as the mean ± standard deviation (SD). For in vivo experiments, 95% confidence intervals (CIs) were calculated to quantify the magnitude of group differences. Prior to statistical testing, the data were assessed for normality and homogeneity of variance. One-way analysis of variance (ANOVA) followed by Tukey’s post hoc test was performed for multiple comparisons. Statistical significance was defined as * *p* < 0.05, ** *p* < 0.01, and ns indicates no significant difference. Graphs and statistical analyses were conducted via R software (version 4.5.1; R Foundation for Statistical Computing, Vienna, Austria), GraphPad Prism 9.5 (GraphPad Software, San Diego, CA, USA), and OriginPro 2023 (OriginLab, Northampton, MA, USA). All experiments were performed with at least three independent biological replicates.

## 3. Results

### 3.1. Isolation and Purification of EPS

Crude EPS was isolated from *E. faecium* HDRsEf1 via trichloroacetic acid and anhydrous ethanol precipitation, yielding 347.7 ± 2.08 mg/L. The crude EPS was further purified on a DEAE-650 M anion-exchange column via stepwise elution with distilled water and 0.2 M NaCl, resulting in two major fractions, designated EPS-T1 (water-eluted) and EPS-T2 (0.2 M NaCl-eluted) (Figure S1A, Supplementary Materials). After purification, the yields of EPS-T1 and EPS-T2 were 159.08 ± 2.36 mg/L and 68.69 ± 1.14 mg/L, respectively. A phenol–sulfuric acid assay revealed carbohydrate contents of 98.63 ± 0.19% for EPS-T1 and 97.37 ± 0.63% for EPS-T2. EPS-T1 was the predominant fraction and was therefore selected for subsequent structural and functional analyses and applications.

### 3.2. Molecular Weight and Monosaccharide Composition

**Figure 1:**
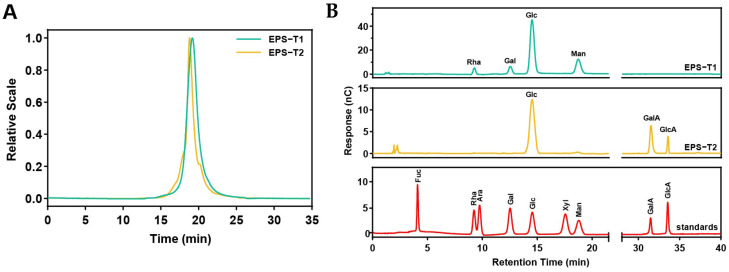
Molecular weights and monosaccharide compositions of EPS-T1 and EPS-T2. (**A**) Molecular weight; (**B**) Monosaccharide composition.

The molecular weight distributions of EPS-T1 and EPS-T2 were determined via HPSEC-MALLS-RI (Figure 1A and Supplementary Materials Table S2). EPS-T1 had a weight-average molecular weight (Mw) of 81.21 ± 1.28 kDa, a number-average molecular weight (Mn) of 19.72 ± 0.76 kDa, and a dispersity (Đ = Mw/Mn) of 4.13 ± 0.14. EPS-T2 exhibited a Mw of 57.79 ± 1.07 kDa, a Mn of 10.06 ± 0.34 kDa, and a dispersity of 5.76 ± 0.11, indicating that both fractions were relatively homogeneous.

The monosaccharide chromatograms displayed clear and well-resolved peaks (Figure 1B and Supplementary Materials Table S3). EPS-T1 consisted of glucose (Glc, 61.86 ± 1.19%), mannose (Man, 24.95 ± 0.95%), galactose (Gal, 7.13 ± 0.18%) and rhamnose (Rha, 6.36 ± 0.11%), with a molar ratio of 9.73:3.92:1.12:1, whereas EPS-T2 contained three monosaccharides, including glucose (Glc, 56.74 ± 1.08%), galacturonic acid (GalA, 34.61 ± 0.67%), and glucuronic acid (GlcA, 8.82 ± 0.22%), with a molar ratio of 6.43:3.92:1, confirming the successful purification and derivatization of the EPS fractions.

### 3.3. FT-IR Spectroscopy and SEM

**Figure 2:**
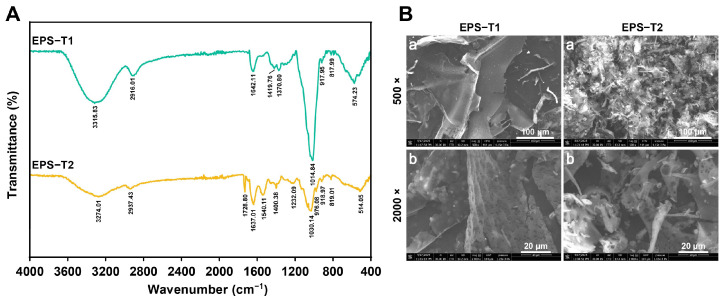
Fourier transform infrared (FT-IR) spectra and scanning electron micrographs (SEM) of EPS-T1 and EPS-T2. (**A**) FT-IR spectra; (**B**) SEM images (a, 500×; b, 2000×).

The FT-IR spectra of EPS-T1 and EPS-T2 revealed typical polysaccharide features (Figure 2A). Both exhibited broad –OH bands at 3315.83 (EPS-T1) and 3274.01 cm^−1^ (EPS-T2), corresponding to hydroxyl stretching vibrations, and C-H stretching peaks at 2916.01 cm^−1^ (EPS-T1) and 2937.43 cm^−1^ (EPS-T2) [10,34]. EPS-T2 showed distinct absorption peaks at 1728.80 cm^−1^ (C=O) and 1540.11 cm^−1^ (COO^−^), indicating its acidic polysaccharide nature, whereas these peaks were absent in EPS-T1, confirming that it is a neutral polysaccharide [27]. Th strong absorption peaks at 1014.84 cm^−1^ (EPS-T1) and 1030.14 cm^−1^ (EPS-T2) corresponded to C–O and C–O–C glycosidic linkages [10,35]. Peaks in the 917–819 cm^−1^ range corresponded to β- and α-pyranose ring structures, confirming the polysaccharide backbone [27].

Scanning electron microscopy (SEM) revealed distinct microstructural differences between EPS-T1 and EPS-T2 (Figure 2B). At 500× magnification, EPS-T1 exhibited relatively smooth, dense, and sheet-like structures, whereas EPS-T2 displayed a fragmented and less organized structure. At 2000× magnification, the surface of EPS-T1 exhibited a porous microstructure with a heterogeneous texture. In contrast, EPS-T2 contained interlaced granular aggregates and larger flocculent clusters. These morphological distinctions indicate different molecular packing and self-assembly behaviors, which may influence their structural stability and functional properties.

### 3.4. Thermal Analysis (TG and DSC)

**Figure 3:**
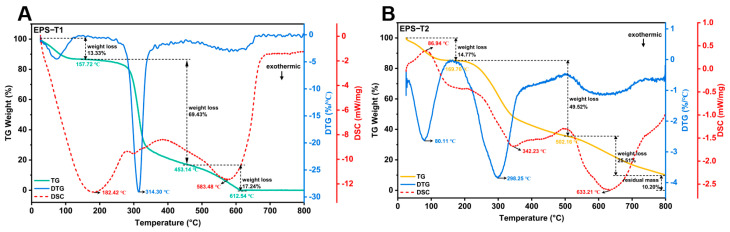
Thermogravimetric (TG) analysis. (**A**) EPS-1; (**B**) EPS-T2.

The thermal properties of EPS-T1 and EPS-T2 were comprehensively evaluated via TG, DTG, and DSC (Figure 3A,B). Both EPS fractions exhibited a characteristic three-step thermal decomposition pattern. The initial weight loss below 200 °C, attributed to the evaporation of bound water, accounted for 13.33% and 14.77% of EPS-T1 and EPS-T2, respectively. The second stage (200–500 °C) represents the main decomposition phase. EPS-T1 had a degradation temperature of 314.30 °C with a weight loss of 69.43%, whereas EPS-T2 decomposed at 298.25 °C with 49.52% weight loss, indicating that EPS-T1 possesses slightly greater thermal stability. The third stage (>500 °C) corresponded to carbonization and structural rearrangements, with EPS-T1 nearly fully decomposed at approximately 612.54 °C, whereas EPS-T2 retained approximately 10.20% of the residue at 800 °C. The DSC curves revealed an endothermic peak at 182.42 °C for EPS-T1 and an initial endothermic peak at 86.94 °C for EPS-T2. Although both EPS fractions exhibited typical three-step thermal decomposition profiles, EPS-T1 demonstrated slightly greater thermal stability than EPS-T2.

### 3.5. Antibacterial Activity

**Figure 4:**
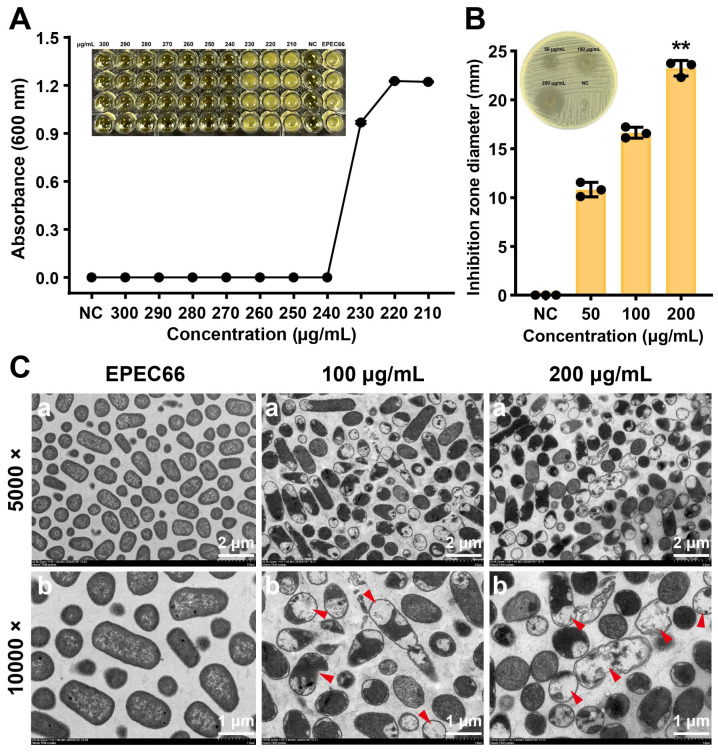
Antibacterial activity of EPS-T1 against *E. coli* EPEC66-lux. (**A**) Minimum inhibitory concentration (MIC) (*n* = 4). (**B**) Agar well diffusion assay (*n* = 3). (**C**) Transmission electron microscopy (TEM) images (a, 5000×; b, 10,000×), plasmolysis, vacuolation, and membrane rupture (red arrows). ** *p* < 0.01.

EPS-T1 exhibited a concentration-dependent inhibitory effect on the growth of *E. coli* EPEC66-lux (Figure 4A). As the concentration of EPS-T1 increased, bacterial growth was progressively suppressed, and complete inhibition was observed at concentrations ≥230 μg/mL, where the OD_600_ values approached those of the blank control, indicating the absence of visible growth. The Oxford cup agar well diffusion assay further confirmed these findings (Figure 4B). The diameter of the inhibition zones increased with increasing EPS-T1 concentration, significantly increasing at 50, 100, and 200 μg/mL EPS-T1, reaching 23.23 ± 0.79 mm at 200 μg/mL EPS-T1, which was significantly greater than that of the negative control (NC, *p* < 0.01).

Transmission electron microscopy (TEM) revealed profound structural damage in bacteria following EPS-T1 treatment. Negative staining TEM revealed structural alterations in *E. coli* EPEC66-lux following EPS-T1 treatment (Supplementary Materials Figure S3). Untreated bacteria exhibited typical rod-shaped structures with intact and well-defined boundaries, dense cytoplasm, and visible pili. In contrast, bacteria treated with EPS-T1 (100 and 200 μg/mL) presented uneven cytoplasmic density and deformation, indicating structural damage. These findings were further corroborated by ultrathin sectioning (Figure 4C), which showed intact cytoplasmic membranes and compact cytoplasm in control EPEC66-lux bacteria. EPS-T1-treated bacteria exhibited pronounced plasmolysis, intracellular vacuolation, and partial rupture of the bacterial membrane, suggesting bacteria lysis. Collectively, these findings indicate that EPS-T1 exerts potent antibacterial activity by simultaneously damaging the bacterial envelope and disrupting the intracellular ultrastructure, ultimately leading to loss of cell integrity and death.

### 3.6. Biofilm Inhibitory and Disruption Activities

**Figure 5:**
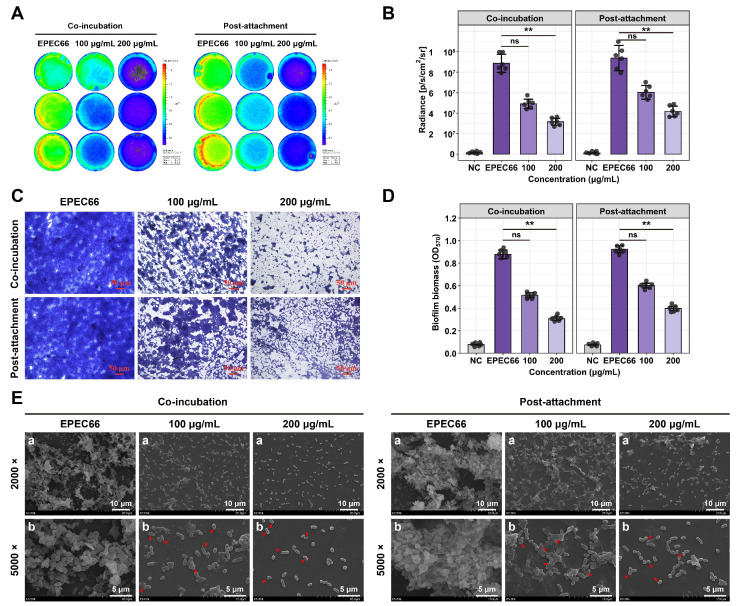
Antibiofilm activity of EPS-T1 against *E. coli* EPEC66-lux (*n* = 4). (**A**) Bioluminescence imaging of biofilms. (**B**) Quantification of bioluminescence intensity. (**C**) Optical microscopy images of crystal violet-stained biofilms (200×). (**D**) Quantification of biofilm formation via crystal violet staining (OD_570_). (**E**) Scanning electron microscopy (SEM) images of biofilms (a, 2000×; b, 5000×), bacterial deformation, surface shrinkage, and collapse (red arrows). ns, not significant; ** *p* < 0.01.

EPS-T1 effectively inhibits initial biofilm formation and disrupts preformed mature biofilms of *E. coli* EPEC66-lux. Biofilm formation and bacterial colonization were assessed via bioluminescence imaging and intensity analysis (Figure 5A,B). Treatment with EPS-T1 reduced bacterial colonization and biofilm coverage in a dose-dependent manner, with a significant reduction observed at 200 μg/mL EPS-T1 (*p* < 0.01). Crystal violet staining further confirmed these results, and optical microscopy revealed that EPS-treated biofilms presented a looser structure and decreased cell density (Figure 5C). Quantitative analysis revealed that EPS-T1 inhibited initial biofilm formation by 41.37 ± 2.02% and 64.93 ± 2.91% at 100 and 200 μg/mL, respectively, and disrupted mature biofilms by 34.93 ± 0.18% and 56.84 ± 0.12%, indicating a dose-dependent inhibitory effect on biofilm biomass (Figure 5D). SEM revealed that EPS-T1 treatment led to a sparse biofilm architecture, reduced bacterial density, and diminished extracellular polymeric substances (Figure 5E). Some bacteria exhibited morphological alterations, including rounding, surface wrinkling, and collapse. These observations indicate that EPS-T1 disrupts biofilm formation and compromises biofilm structural integrity, highlighting its potential as an antibiofilm agent.

### 3.7. Cell Viability

**Figure 6:**
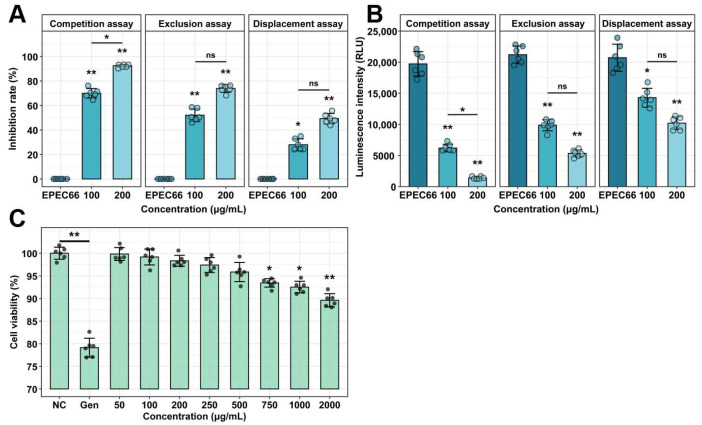
Cytotoxicity and antiadhesion activity of EPS-T1 (*n* = 6). (**A**) Inhibition rate of *E. coli* EPEC66-lux adhesion to IPI-2I cells. (**B**) Luminescence intensity reflecting EPEC66-lux adhesion to IPI-2I cells. (**C**) Cytotoxicity of IPI-2I cells by CCK-8 assay. ns, not significant; * *p* < 0.05; ** *p* < 0.01.

The effect of EPS-T1 on IPI-2I cell viability was assessed via the CCK-8 assay (Figure 6C). No significant changes were observed at 0–500 μg/mL (*p* > 0.05). A slight decrease was observed at 750 μg/mL (*p* < 0.05), and more pronounced reductions were observed at 1000 and 2000 μg/mL (*p* < 0.01). However, the cell viability remained at 89.60 ± 1.47% even at the highest concentration, indicating that EPS-T1 has low cytotoxicity. In contrast, the positive control gentamicin (100 μg/mL) reduced cell viability to 79.13 ± 2.09%.

### 3.8. Inhibitory Effects on Adhesion

EPS-T1 significantly inhibited the adhesion of *E. coli* EPEC66-lux to IPI-2I cells under competitive, exclusion, and displacement conditions (Figure 6A,B). In the competition assay, adhesion was reduced by 70.00 ± 1.15% and 92.48 ± 0.81% at 100 and 200 μg/mL EPS-T1, respectively. In the exclusion experiment, the inhibition rates were 52.10 ± 2.09% and 73.93 ± 1.04%; in the displacement assay, they were 27.94 ± 1.61% and 49.27 ± 1.19%, respectively. The fluorescence intensity results were consistent with the colony counts, confirming the dose-dependent antiadhesion effect.

### 3.9. Antioxidant Activity

**Figure 7:**
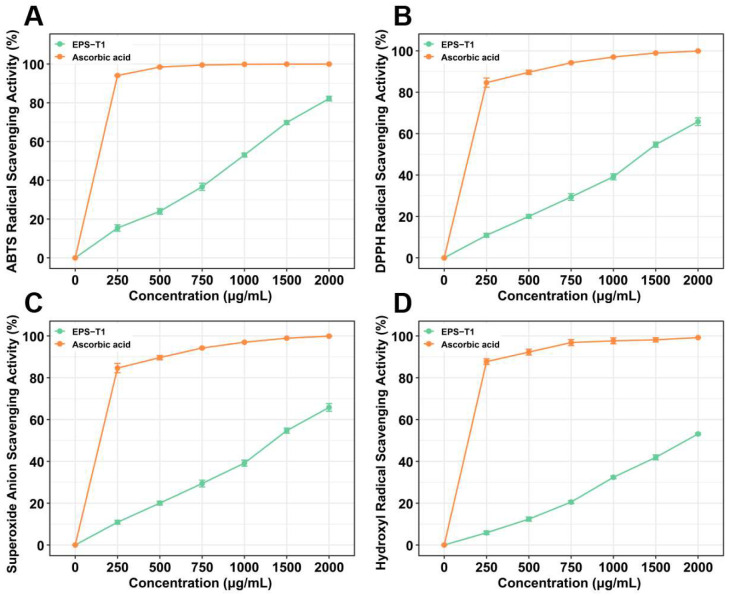
Antioxidant activities of EPS-T1 (*n* = 6). (**A**) ABTS radical scavenging activity. (**B**) DPPH radical scavenging activity. (**C**) Superoxide anion radical scavenging activity; (**D**) Hydroxyl radical scavenging activity.

EPS-T1 exhibited dose-dependent antioxidant activity at concentrations ranging from 250~2000 μg/mL. In the ABTS assay (Figure 7A), the scavenging rate increased from 15.38 ± 1.63% at 250 μg/mL to 82.15 ± 1.22% at 2000 μg/mL. In the DPPH assay (Figure 7B), the maximum scavenging activity reached 65.80 ± 1.85% at 2000 μg/mL. In the superoxide anion and hydroxyl radical assays (Figure 7C,D), the maximum scavenging rates at 2000 μg/mL were 59.94 ± 0.60% and 53.13 ± 0.43%, respectively, indicating a stable antioxidant potential across different radical systems.

### 3.10. Animal Results

#### 3.10.1. Body Weight Changes

**Figure 8:**
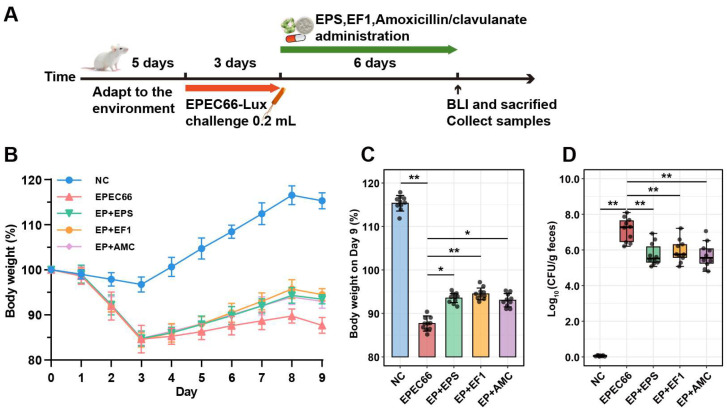
Protective effects of EPS-T1 in an EPEC-infected mouse model. (**A**) Schematic of the infection and treatment protocol. (**B**,**C**) Body weight changes in the mice (*n* = 10). (**D**) qPCR analysis of the intestinal bacterial load in the mice (*n* = 10). * *p* <0.05; ** *p* < 0.01.

Body weight and clinical symptoms were monitored daily to evaluate the effects of *E. coli* EPEC66-lux infection and the therapeutic efficacy of EPS-T1 (Figure 8B,C). After 3 days of infection, mice infected with EPEC66-lux presented transient soft stools and a significant reduction in body weight compared with those of the NC group, without reaching humane endpoints, confirming the detrimental impact of EPEC infection on host growth. Compared with those of the EPEC66 group, the body weights of all the treated groups recovered after 5 days of treatment. Among the treatment groups, the EP + EF1 group exhibited pronounced improvement, with the final body weight increased by 7.78% (95% CI [6.22, 9.33], *p* < 0.01), relative to the EPEC66 group. Similarly, the EP + EPS and EP + AMC groups presented increases of 6.66% (95% CI [5.23, 8.09], *p* < 0.05) and 6.11% (95% CI [4.47, 7.75], *p* < 0.05), respectively. These results suggest that EPS-T1 effectively alleviated EPEC-induced growth inhibition and contributed to body weight recovery in infected mice.

#### 3.10.2. In Vivo Colonization and Intestinal Bacterial Load of EPEC66-Lux

**Figure 9:**
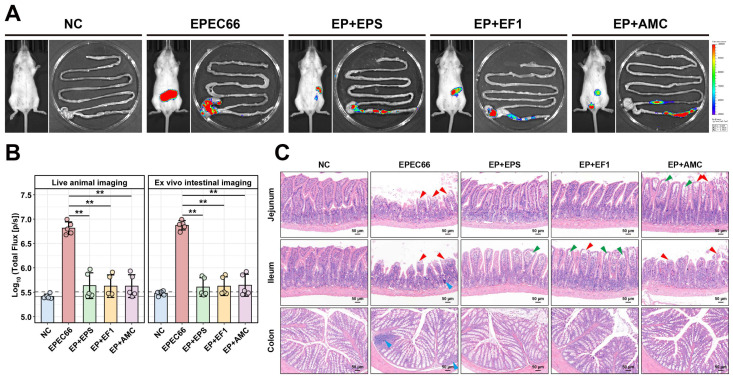
In vivo imaging and histopathological evaluation of intestinal tissues in mice. (**A**) In vivo and intestinal bioluminescence images of the mice infected with EPEC66-lux; representative images are shown; pseudocolor heatmaps indicate the bioluminescence intensity from low (blue) to high (red). (**B**) Quantification of the abdominal and intestinal bioluminescence intensity from the mice in the experiment illustrated in A. (*n* = 5), gray lines indicate detection thresholds, determined as the mean (solid line) and mean + 2 SDs (dashed line) of background bioluminescence from the control uninfected mice. (**C**) Representative H&E-stained sections of the intestine (scale bar 50 μm), epithelial cell shedding, exposure of the lamina propria (red arrows), separation of the mucosal epithelium from the lamina propria (green arrows), and localized lymphocytic aggregation (blue arrows). ** *p* < 0.01.

To evaluate the in vivo antibacterial effect of EPS-T1, the colonization of EPEC66-lux-infected mice in the intestinal tract was assessed via IVIS imaging and qPCR. As shown in Figure 9A (Figure S4, Supplementary Materials), the mice in the EPEC66 group presented strong bioluminescence signals in the abdomen, indicating persistent colonization of EPEC66-lux in the gut, whereas the signals in the EP + EPS, EP + EF1, and EP + AMC groups were markedly reduced, with luminescence primarily localized to the cecum and colon. Quantitative bioluminescence intensity analysis (Figure 9B) revealed that both systemic and intestinal luminescence signals in the EP + EPS, EP + EF1, and EP + AMC groups were significantly lower than those in the EPEC66 group (*p* < 0.01), with EPS-T1 exhibiting inhibitory effects comparable to those of the probiotic and antibiotic treatments. Further quantification of the EPEC load in the intestinal contents via qPCR (Figure 8D) revealed that the bacterial abundance in the EP + EPS, EP + EF1 and EP + AMC groups was significantly lower than that in the EPEC66 group (*p* < 0.01), with reductions of 1.38 log_10_ CFU/g (95% CI [0.77, 2.10]), 1.19 log_10_ CFU/g (95% CI [0.57, 1.81]) and 1.41 log_10_ CFU/g (95% CI [0.73, 2.08]), respectively, consistent with the imaging data. These results indicate that EPS-T1 effectively inhibits the intestinal colonization of *E. coli*.

#### 3.10.3. Histopathological Analyses

Histological analysis and scoring of H&E-stained sections (Figure 9C and Supplementary Materials Figure S5) revealed significant pathological damage in the jejunum, ileum, and colon following EPEC66-lux infection, characterized by villus atrophy, epithelial cell shedding, and exposure of the lamina propria, and was accompanied by localized lymphocytic aggregation. Similarly, the histopathological score of the EPEC66-lux group was significantly greater than that of the NC group (*p* < 0.01). In contrast, the EP + EPS group exhibited markedly attenuated intestinal injury, with well-preserved villus architecture, only minor separation of the mucosal epithelium from the lamina propria, and no apparent necrosis or inflammatory infiltration. The histopathological score in the EP + EPS group was significantly lower than that in the EPEC66-lux group (*p* < 0.05), indicating improved mucosal integrity. These observations indicate that EPS-T1 contributes to maintaining the intestinal structure and mitigating EPEC-induced mucosal damage.

#### 3.10.4. Inflammatory Cytokine Levels

**Figure 10:**
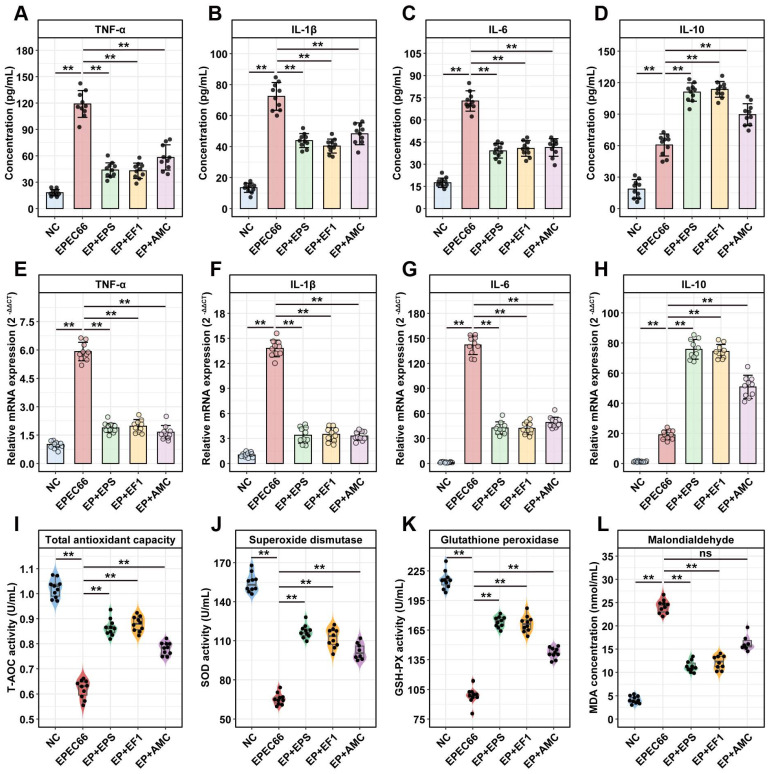
Anti-inflammatory and antioxidant effects in mice. (**A**–**D**) Protein levels of the inflammatory cytokines TNF-α, IL-6, IL-1β and IL-10 in ileum tissues measured via ELISA (*n* = 10); (**E**–**H**) mRNA expression levels of the inflammatory cytokines TNF-α, IL-1β, IL-6 and IL-10 in ileum tissues measured via qPCR (*n* = 10); (**I**–**L**) Antioxidant enzyme activity (T-AOC, SOD, and GSH-PX) and lipid peroxidation (MDA) levels in the serum (*n* = 10). ns, not significant; ** *p* < 0.01.

The ELISA results (Figure 10A–D) revealed that EPEC66 infection significantly increased the levels of the pro-inflammatory cytokines TNF-α, IL-1β, and IL-6 in the ileum, compared with those in the NC group (*p* < 0.01). Following EPS-T1 treatment (EP + EPS group), the ileal levels of pro-inflammatory cytokines TNF-α, IL-1β and IL-6 were significantly lower than those in the EPEC66 group (*p* < 0.01), decreasing to 0.37-fold (95% CI [0.22, 0.51]), 0.53-fold (95% CI [0.40, 0.67]) and 0.61-fold (95% CI [0.46, 0.75]), respectively. In contrast, the level of the anti-inflammatory cytokine IL-10 was significantly increased, reaching 1.83-fold (95% CI [1.76, 1.91], *p* < 0.01). The qPCR results (Figure 10E–H) revealed a similar trend, which was consistent with the ELISA results. Probiotic and antibiotic treatments also modulated cytokine levels, with EPS-T1 showing efficacy comparable to that of the EP + EF1 group. These results indicate that EPS-T1 effectively modulates ileal cytokine levels, attenuating EPEC66-induced inflammatory responses.

#### 3.10.5. Antioxidant Activity in Serum

As shown in Figure 10I–L, EPEC66 infection induced a pronounced oxidative stress response in the mice, as evidenced by significantly decreased serum activities of T-AOC, SOD, and GSH-PX (*p* < 0.01) and markedly increased MDA levels (*p* < 0.01) compared with those in the NC group. Treatment with EPS-T1 (EP + EPS group) significantly improved the systemic antioxidant status of EPEC66-infected mice. The serum T-AOC, GSH-PX, and SOD activities increased 1.39-fold (95% CI [1.36, 1.43]), 1.71-fold (95% CI [1.67, 1.76]), and 1.75-fold (95% CI [1.72, 1.79]) in the EPEC66 group (*p* < 0.01), respectively, whereas the MDA level decreased 0.47-fold (95% CI [0.37, 0.56]), *p* < 0.01), indicating that EPS-T1 effectively alleviated oxidative stress and restored antioxidant capacity.

## 4. Discussion

This study systematically analyzed the physicochemical properties and biological activities of EPS produced by *E. faecium* HDRsEf1, with a focus on the major fraction, EPS-T1. The results demonstrated that EPS-T1 exhibited significant antibacterial, antibiofilm, antiadhesive, and antioxidant activities. In a mouse EPEC infection model, EPS-T1 effectively alleviated intestinal damage, as evidenced by reduced bacterial colonization, amelioration of gut mucosal damage, and alleviation of inflammatory responses. These findings highlight EPS-T1 as a multifunctional, probiotic-derived biopolymer with potential applications in the prevention and management of intestinal infections.

The elution profile of the crude EPS exhibited multiple peaks, indicating compositional complexity and the potential presence of polysaccharides with distinct molecular characteristics. The crude EPS yield reached 347.7 ± 2.08 mg/L, which was greater than that previously reported for *E. faecium* MW725386 (257.32 ± 31.65 mg/L) [14] and *E. faecium* (155 mg/L) [36], and comparable to that reported for *E. faecium* K1 (355 ± 0.019 mg/L) [37]. Such variations among strains are generally attributed to differences in growth media, environmental conditions, and genetic background [38], highlighting EPS production potential for practical applications. After purification, EPS displayed a single symmetrical peak with molecular weights ranging from 57 to 81 kDa, within the reported range for *Enterococcus*-derived EPS (2.5 × 10^4^ to 1.0 × 10^8^ Da) [9,39]. Low-molecular-weight EPS has been suggested to exhibit enhanced biological activity because of improved solubility and accessibility of functional groups [15].

*Enterococcus*-derived EPS exhibit diverse monosaccharide compositions, typically dominated by glucose, galactose, mannose, fructose, arabinose, xylose, and rhamnose, which are closely associated with antimicrobial, antioxidant, and immunomodulatory properties [15,40,41]. EPS-T1 in this study displayed a similar monosaccharide composition, suggesting that its potential antimicrobial and antioxidant activities may be linked to structural features. The functional groups of EPS influence its physicochemical and biological properties, including viscosity, antimicrobial activity, and antioxidant potential [42,43]. As a neutral polysaccharide lacking charged groups, EPS-T1 likely exerts its bioactivity through molecular conformation and flexibility rather than electrostatic interactions, which may enable the regulation of microbial adhesion and host immune responses via steric hindrance and nonspecific interactions [38,42]. The differences in the FT-IR spectra of EPS from various *Enterococcus* strains further indicate strain-specific structural and physicochemical variations [37].

Microstructural analysis revealed that EPS-T1 has a porous, sheet morphology with locally compact surfaces, which enhances its water solubility, structural stability, and interactions with target molecules, thereby improving its antioxidant activity and bioavailability [44]. The compact surface may also inhibit bacterial aggregation and colonization [24]. Thermal analysis revealed that EPS-T1 exhibited moderate decomposition and a relatively low residual mass, reflecting good thermal adaptability. These properties support potential industrial feed applications and suggest that EPS-T1 can maintain structural integrity and functionality during gastrointestinal transit [13].

In this study, EPS-T1 exhibited remarkable antibacterial activity, demonstrating concentration-dependent inhibition of *E*. *coli* growth. These findings are consistent with previous reports, where EPS derived from probiotics demonstrated notable inhibitory effects against both Gram-positive and Gram-negative bacteria. For example, EPS-MS79 from *E. faecium* strongly inhibited *S. aureus*, *S. typhimurium*, *L. monocytogenes*, and *E. coli* [9], whereas EPS from lactic acid bacteria inhibited *E. coli*, *E. faecalis*, and *L. monocytogenes* [45]. The antibacterial mechanism involves disruption of cell wall integrity, inhibition of peptidoglycan biosynthesis, and competitive blockade of sugar uptake, causing impaired nutrient acquisition and cell lysis [46]. EPSs also limit bacterial translocation and promote beneficial gut microbes such as *Lactobacillus* and *Bifidobacterium* [18].

With respect to antibiofilm, EPS-T1 effectively impeded both the initial adhesion and maturation of *E. coli* biofilms in a dose-dependent manner. This finding is similar to the inhibitory effects of EPS from *R. mucilaginosa* on the biofilms of *S. aureus*, *E. coli*, and *P. aeruginosa* in a concentration-dependent manner [46] and is consistent with the reported effects of *E. faecium* EPS on the biofilm formation of Gram-positive pathogens, including *L. monocytogenes*, *S. aureus, E. faecalis*, and *S. typhi* [37,47]. The antibiofilm mechanism may involve the modulation of bacterial surface properties, competition with adhesion proteins, and interference with signal transduction pathways, which prevent initial attachment, cell–cell interactions, and the downregulation of biofilm-associated gene expression [48,49]. These findings indicate that EPS-T1 not only exerts direct antibacterial effects but also enhances its in vitro functional activity via antibiofilm mechanisms.

In addition to their antibacterial and antibiofilm effects, EPSs are widely recognized for their ability to inhibit pathogen adhesion to intestinal epithelial cells, which represents a key indicator of probiotic functionality. EPS from *L. reuteri* reduces ETEC adhesion to IPEC-1 cells [50], and EPS extracted from fermented SoyD inhibits ETEC K88 colonization of porcine mucin [51]. EPS from *E. faecium* inhibited *L. monocytogenes*, *S. aureus*, and *S. enterica* colonization of HCT-15 and HT-29 cells in exclusion, competition, and displacement assays [28,39]. Consistent with previous reports, EPS-T1 produced by HDRsEf1 significantly reduced EPEC adhesion to intestinal epithelial cells, confirming its antiadhesive activity. The antiadhesion effect likely results from competitive occupation of host cell binding sites, thereby preventing pathogen attachment and reducing bacterial colonization and infection [52,53], highlighting the protective potential of EPS in vivo and its role in interfering with pathogen–host interactions.

EPSs generally exhibit concentration-dependent radical scavenging activity [54]. In this study, EPS-T1 showed broad-spectrum radical scavenging capacity, which is in line with reports on EPS from *E. faecalis* and *E. hirae*, which are known to possess strong DPPH, superoxide, and hydroxyl radical scavenging activities [55,56]. The antioxidant potential of EPS is closely related to its structural features, monosaccharide composition, and amorphous structure, all of which facilitate electron donation and free radical neutralization [57]. Considering that oxidative stress plays a pivotal role in maintaining host homeostasis and is closely linked to intestinal inflammation and epithelial injury, the antioxidant potential of EPS-T1 may contribute significantly to its protective effects on gut health.

*E. coli* infection is known to impair intestinal barrier integrity and induce inflammation, leading to reduced nutrient absorption and growth [58]. In this study, EPS supplementation mitigated the negative impact of EPEC on body weight, which aligns with previous evidence that EPS with prebiotic and immunomodulatory properties can improve nutrient utilization and promote growth recovery [59,60]. Although transient body weight decreases were observed, these decreases may largely result from 12 h fasting periods before infection and sacrifice, as similar fluctuations have been documented in related animal models [61,62].

In terms of intestinal barrier protection, HE staining revealed that EPS-T1 significantly alleviated villus atrophy and epithelial necrosis in EPEC-challenged mice, suggesting its potential role in maintaining intestinal structural integrity. This observation is consistent with previous reports that EPS derived from LAB exert barrier-protective effects. EPS produced by *L. plantarum* NMGL2 restored epithelial integrity in DSS-induced colitis, increasing the expression of tight junction proteins ZO-1 and occludin and suppressing NF-κB p65 activation [63]. Similarly, purified EPS from *S. thermophilus* protected intestinal barrier integrity by upregulating claudin-1, occludin, and E-cadherin, and alleviating DSS-induced epithelial damage [64]. These findings support that LAB-derived EPSs can reinforce tight junctions and mitigate inflammation-mediated barrier disruption. The histological improvement observed after EPS-T1 treatment may involve similar mechanisms, which warrants further investigation.

An imbalance between pro-inflammatory and anti-inflammatory cytokines can lead to enhanced inflammatory responses and immune dysfunction [63]. In the present study, we measured the levels of TNF-α, IL-1β, and IL-10 in ileal tissues and found that EPS treatment significantly (*p* < 0.01) reduced TNF-α, IL-1β, and IL-6 levels, while markedly increasing IL-10 levels. Similarly, previous studies have shown that EPS produced by *L. plantarum* can decrease TNF-α, IL-1β, and IL-6 levels, inhibit NO production, and increase IL-10 expression [16,63]. Gao et al. reported that *L. rhamnosus* EPS alleviates *S. typhimurium*-induced intestinal inflammation in mice, reducing the levels of pro-inflammatory (TNF-α and IL-1β) and increasing the levels of the anti-inflammatory cytokine IL-10, thereby contributing to immune homeostasis and exerting protective effects in intestinal disease models [65]. Notably, such immunomodulatory effects often occur synergistically with improved barrier function. Previous studies have indicated that EPS maintains host immune homeostasis by suppressing the expression of key proteins in the TLR4/NF-κB/MAPK signaling pathway [63,65,66]. Collectively, these results imply that EPS-T1 might participate in regulating the immune epithelial balance, thereby contributing to intestinal health, although the precise molecular mechanisms require further investigation

Oxidative stress plays a critical role in intestinal inflammation and pathogen invasion, as high levels of reactive oxygen species (ROS) can compromise the intestinal epithelial barrier and amplify inflammatory signaling [16]. The data from this study indicate that EPS-T1 significantly increased serum SOD, GSH-PX, and T-AOC activity in mice while reducing MDA levels, which may contribute to the alleviation of intestinal inflammation and oxidative stress. Similarly, EPS derived from *L. fermentum* and *L. rhamnosus* increased T-AOC and SOD activity while decreasing MDA levels in nematodes [66,67]; EPS from *L. plantarum* also alleviated DSS-induced oxidative stress in mice by increasing GSH and SOD levels while significantly reducing nitric oxide (NO) and MDA levels [63]. These antioxidant effects may be attributed to the free radical scavenging capacity of EPS and its ability to regulate redox homeostasis [11,68]. Nonetheless, the precise mechanistic link between these in vitro effects and the observed in vivo protection warrants further investigation.

Although this study provides valuable insights into the structural features, in vitro bioactivities, and protective effects of EPS-T1 in a mouse model, limitations should be noted. The current work focused on short-term, EPEC-induced acute intestinal inflammation, and the long-term efficacy and protective potential of EPS-T1 remain to be clarified. Moreover, whether similar protective effects can be observed against other enteric pathogens or across different animal species warrants further validation. Although in vitro assays confirmed antibacterial, antibiofilm, and antioxidant activities, the mechanistic link between these effects and the observed in vivo benefits remains to be fully elucidated. In addition, the physicochemical stability of EPS-T1 under industrial conditions (e.g., heat, pH fluctuations, and storage) and its scalability for large-scale production have yet to be evaluated. Addressing these limitations in future studies will be crucial for elucidating the functional mechanisms of EPS-T1 and advancing its translational potential in food, feed, and biomedical applications

## 5. Conclusions

This study systematically evaluated the structural characteristics, in vitro activities, and in vivo effects of EPS-T1 derived from *E. faecium* HDRsEf1. The results revealed that EPS-T1 is a neutral heteropolysaccharide with a porous, sheet-like microstructure and high thermal stability, exhibiting significant antibacterial, antibiofilm, antioxidant, and antiadhesive activities in vitro. In a mouse EPEC infection model, EPS-T1 effectively reduced bacterial colonization, alleviated intestinal tissue damage, downregulated pro-inflammatory cytokines, upregulated anti-inflammatory cytokines, enhanced antioxidant defense, and improved intestinal barrier function. These findings highlight the close association between the structural features of EPS-T1 and its biological functions, emphasizing the importance of structure–function relationships in microbial EPS. Overall, EPS-T1 represents a promising multifunctional biopolymer that may contribute to health management and disease prevention, offering a theoretical basis for its further application.

## Data Availability

The data presented in this study are available upon request from the corresponding author.

## Supplementary Materials

The following supporting information can be downloaded at https://www.mdpi.com/article/10.3390/nu17233667/s1. Figure S1. Characterization of EPS fractions. (A) Elution profiles of crude EPS on DEAE-650 M anion-exchange column. (B) Calibration curve for total sugar content determined by the phenol–sulfuric acid method (glucose as standard). Figure S2. Standard curve constructed based on the *luxA* gene for quantification of bacterial load. Figure S3. Negative staining TEM images of bacteria (a, 5000×; b, 10,000×). Figure S4. In vivo and intestinal bioluminescence imaging of mice infected with EPEC66-lux. Figure S5. Histologic damage score (*n* = 5). Table S1. Primers for detecting inflammation. Table S2. Molecular weight distributions. Table S3. Monosaccharide composition (%).

## Abbreviations

The following abbreviations are used in this manuscript:

EPS	Exopolysaccharides
LAB	Lactic acid bacteria
Ara	Arabinose
Fuc	Fucose
Gal	Galactose
GalA	Galacturonic acid
Glc	Glucose
Man	Mannose
Rha	Rhamnose
Xyl	xylose
CFU	Colony-forming units
MIC	Minimum inhibitory concentration
MOI	Multiplicity of infection
H&E	Staining hematoxylin-eosin staining
GSH-PX	Glutathione peroxidase
MDA	Malondialdehyde
SOD	Superoxide dismutase
T-AOC	Total antioxidant capacity
NO	Nitric oxide
TNF-α	Tumor necrosis factor-α
IL-6	Interleukin-6
IL-10	Interleukin-10
IL-1β	Interleukin-1β
TCA	Trichloroacetic acid

## References

[1] Farré R., Fiorani M., Abdu Rahiman S., Matteoli G. (2020). Intestinal Permeability, Inflammation and the Role of Nutrients. Nutrients.

[2] Brodin P. (2022). Immune-microbe interactions early in life: A determinant of health and disease long term. Science.

[3] Beraldo L.G., Borges C.A., Maluta R.P., Cardozo M.V., de Ávila F.A. (2023). Molecular analysis of enteropathogenic *Escherichia coli* (EPEC) isolates from healthy food-producing animals and humans with diarrhoea. Zoonoses Public Health.

[4] Arais L.R., Barbosa A.V., Andrade J.R.C., Gomes T.A.T., Asensi M.D., Aires C.A.M., Cerqueira A.M.F. (2018). Zoonotic potential of atypical enteropathogenic *Escherichia coli* (aEPEC) isolated from puppies with diarrhoea in Brazil. Vet. Microbiol..

[5] Lee J.B., Kim S.K., Yoon J.W. (2022). Pathophysiology of enteropathogenic *Escherichia coli* during a host infection. J. Vet. Sci..

[6] Clarke S.C., Haigh R.D., Freestone P.P., Williams P.H. (2003). Virulence of enteropathogenic *Escherichia coli*, a global pathogen. Clin. Microbiol. Rev..

[7] Zhang S., Wu Q., Zhang J., Zhu X. (2016). Occurrence and Characterization of Enteropathogenic *Escherichia coli* (EPEC) in Retail Ready-to-Eat Foods in China. Foodborne Pathog. Dis..

[8] Wang W., Ju Y., Liu N., Shi S., Hao L. (2023). Structural characteristics of microbial exopolysaccharides in association with their biological activities: A review. Chem. Biol. Technol. Agric..

[9] Ayyash M., Stathopoulos C., Abu-Jdayil B., Esposito G., Baig M., Turner M.S., Baba A.S., Apostolopoulos V., Al-Nabulsi A., Osaili T. (2020). Exopolysaccharide produced by potential probiotic *Enterococcus faecium* MS79: Characterization, bioactivities and rheological properties influenced by salt and pH. LWT.

[10] Tan X., Ma B., Wang X., Cui F., Li X., Li J. (2024). Characterization of Exopolysaccharides from *Lactiplantibacillus plantarum* PC715 and Their Antibiofilm Activity Against *Hafnia alvei*. Microorganisms.

[11] Sharma P., Sharma A., Lee H.-J. (2024). Antioxidant potential of exopolysaccharides from lactic acid bacteria: A comprehensive review. Int. J. Biol. Macromol..

[12] Xu Q., Wang M.-M., Li X., Ding Y.-R., Wei X.-Y., Zhou T. (2024). Antioxidant and anti-inflammatory activities and action mechanisms of exopolysaccharides from *Lactiplantibacillus plantarum* Z-1. Food Biosci..

[13] Zhang X., Zhang C., Xiao L., Zhao X., Ma K., Ji F., Azarpazhooh E., Ajami M., Rui X., Li W. (2024). Digestive characteristics of extracellular polysaccharide from *Lactiplantibacillus plantarum* T1 and its regulation of intestinal microbiota. Int. J. Biol. Macromol..

[14] Tarique M., Ali A.H., Kizhakkayil J., Liu S., Oz F., Dertli E., Kamal-Eldin A., Ayyash M. (2024). Exopolysaccharides from *Enterococcus faecium* and *Streptococcus thermophilus*: Bioactivities, gut microbiome effects, and fermented milk rheology. Food Chem. X.

[15] Kavitake D., Devi P.B., Delattre C., Reddy G.B., Shetty P.H. (2023). Exopolysaccharides produced by *Enterococcus* genus—An overview. Int. J. Biol. Macromol..

[16] Huang Y., Liang W., Lu Y., Xiong J., Liu D., Jia X. (2025). Identification, Antioxidant and Immunomodulatory Activities of a Neutral Exopolysaccharide from *Lactiplantibacillus plantarum* DMDL 9010. Nutrients.

[17] Du R., Yu L., Yu N., Ping W., Song G., Ge J. (2022). Characterization of exopolysaccharide produced by *Levilactobacillus brevis* HDE-9 and evaluation of its potential use in dairy products. Int. J. Biol. Macromol..

[18] Ding C., Wu H., Cao X., Gao Z., Tang Z., Fan W., Yan L., Liu B., Lin H., Song S. (2022). *Lactobacillus crispatus*-derived exopolysaccharides with antibacterial activity limit *Salmonella typhimurium* invasion by inhibiting inflammasome-mediated pyroptosis. Food Funct..

[19] Abedin M.M., Chourasia R., Phukon L.C., Sarkar P., Ray R.C., Singh S.P., Rai A.K. (2024). Lactic acid bacteria in the functional food industry: Biotechnological properties and potential applications. Crit. Rev. Food Sci. Nutr..

[20] Shi D., Xiao Y., Bi D., Xiong Y., Wang X., Gao X., Li Z., Zhou Z., Liu M., Xu Q. (2013). A Beneficial Enterococcus faecium Strain’s Screening and Application.

[21] Zhuo W.X., Zhao Y., Zhao X.L., Yao Z.M., Qiu X.X., Huang Y.X., Li H.X., Shen J., Zhu Z.H., Li T.T. (2023). Enteropathogenic *Escherichia coli* is a predominant pathotype in healthy pigs in Hubei Province of China. J. Appl. Microbiol..

[22] Lv H., Teng Q., Chen J., Peng L., Ren Z., Ma L., Yang W., Yu B., Wu Z., Wan C. (2024). Probiotic potential of a novel exopolysaccharide produced by *Bifidobacterium animalis* subsp. *Lactis* SF. LWT.

[23] Akkerman R., Oerlemans M.M.P., Ferrari M., Fernández-Lainez C., Walvoort M.T.C., de Vos P. (2025). Exopolysaccharides from *Bifidobacterium longum* subsp. infantis and *Bifidobacterium adolescentis* modulate Toll-like receptor signaling. Carbohydr. Polym..

[24] Pramudito T.E., Desai K., Voigt C., Smid E.J., Schols H.A. (2024). Dextran and levan exopolysaccharides from tempeh-associated lactic acid bacteria with bioactivity against enterotoxigenic *Escherichia coli* (ETEC). Carbohydr. Polym..

[25] Tilwani Y.M., Lakra A.K., Domdi L., Yadav S., Jha N., Arul V. (2021). Optimization and physicochemical characterization of low molecular levan from *Enterococcus faecium* MC-5 having potential biological activities. Process Biochem..

[26] Shi Y., Liu J., Zhou H., Wu Z., Qiu Y., Ye C. (2025). Dihydromyricetin alleviates ETEC K88-induced intestinal inflammatory injury by inhibiting quorum sensing-related virulence factors. BMC Microbiol..

[27] Zhao H., Abbas S., Ren J., Huang H., Song Y., Su X., Wu Q., Ma Y., Tang H., Gao Y.Z. (2025). Dextran from human feces-derived *Weissella cibaria* facilitates intestinal mucosal barrier function by modulating gut bacteria and propionate levels. Carbohydr. Polym..

[28] Tilwani Y.M., Lakra A.K., Domdi L., Jha N., Arul V. (2022). Characterization of potential probiotic bacteria *Enterococcus faecium* MC-5 isolated from the gut content of *Cyprinus carpio specularis*. Microb. Pathog..

[29] Jin W., Zhou H., Zhao H., Pei Y., Su F., Li Y., Luo T. (2025). Isolation, In Vitro Antioxidant Capacity, Hypoglycemic Activity and Immunoactivity Evaluation of Polysaccharides from *Coriandrum sativum* L. Antioxidants.

[30] Ledwaba S.E., Costa D.V.S., Bolick D.T., Giallourou N., Medeiros P., Swann J.R., Traore A.N., Potgieter N., Nataro J.P., Guerrant R.L. (2020). Enteropathogenic *Escherichia coli* Infection Induces Diarrhea, Intestinal Damage, Metabolic Alterations, and Increased Intestinal Permeability in a Murine Model. Front. Cell. Infect. Microbiol..

[31] MacArthur Clark J.A., Sun D. (2020). Guidelines for the ethical review of laboratory animal welfare People’s Republic of China National Standard GB/T 35892-2018 [Issued 6 February 2018 Effective from 1 September 2018]. Animal Model Exp Med..

[32] Wong D.V., Lima-Júnior R.C., Carvalho C.B., Borges V.F., Wanderley C.W., Bem A.X., Leite C.A., Teixeira M.A., Batista G.L., Silva R.L. (2015). The Adaptor Protein Myd88 Is a Key Signaling Molecule in the Pathogenesis of Irinotecan-Induced Intestinal Mucositis. PLoS ONE.

[33] Unkovič A., Boštjančič E., Belič A., Perše M. (2023). Selection and Evaluation of mRNA and miRNA Reference Genes for Expression Studies (qPCR) in Archived Formalin-Fixed and Paraffin-Embedded (FFPE) Colon Samples of DSS-Induced Colitis Mouse Model. Biology.

[34] Ren Y., Pei F., Cao X., Zhang W., Du R., Ge J., Ping W. (2023). Purification of exopolysaccharides from *Lactobacillus rhamnosus* and changes in their characteristics by regulating quorum sensing genes via polyphenols. Int. J. Biol. Macromol..

[35] Liu Y., Mao K., Zhang N., Chitrakar B., Huang P., Wang X., Yang B., Sang Y. (2022). Structural characterization and immunomodulatory effects of extracellular polysaccharide from *Lactobacillus paracasei* VL8 obtained by gradient ethanol precipitation. J. Food Sci..

[36] Yuksekdag Z., Ahlatcı N.S., Hajikhani R., Darilmaz D.O., Beyatli Y. (2021). Safety and metabolic characteristics of 17 *Enterococcus faecium* isolates. Arch. Microbiol..

[37] Bhat B., Bajaj B.K. (2018). Hypocholesterolemic and bioactive potential of exopolysaccharide from a probiotic *Enterococcus faecium* K1 isolated from kalarei. Bioresour. Technol..

[38] Venkatesh P., Balraj M., Ayyanna R., Ankaiah D., Arul V. (2016). Physicochemical and biosorption properties of novel exopolysaccharide produced by *Enterococcus faecalis*. LWT.

[39] Jia K., Tao X., Liu Z., Zhan H., He W., Zhang Z., Zeng Z., Wei H. (2019). Characterization of novel exopolysaccharide of *Enterococcus faecium* WEFA23 from infant and demonstration of its in vitro biological properties. Int. J. Biol. Macromol..

[40] Ali A.H., Bamigbade G., Tarique M., Esposito G., Obaid R., Abu-Jdayil B., Ayyash M. (2023). Physicochemical, rheological, and bioactive properties of exopolysaccharide produced by a potential probiotic *Enterococcus faecalis* 84B. Int. J. Biol. Macromol..

[41] Kansandee W., Moonmangmee D., Moonmangmee S., Itsaranuwat P. (2019). Characterization and *Bifidobacterium* sp. growth stimulation of exopolysaccharide produced by *Enterococcus faecalis* EJRM152 isolated from human breast milk. Carbohydr. Polym..

[42] Dong J., Chi Z., Lu S., Xie X., Gong P., Li H., Liu W. (2025). Bacterial exopolysaccharides: Characteristics and antioxidant mechanism. Int. J. Biol. Macromol..

[43] Salimi F., Farrokh P. (2023). Recent advances in the biological activities of microbial exopolysaccharides. World J. Microbiol. Biotechnol..

[44] Wang X., Xu M., Xu D., Ma K., Zhang C., Wang G., Dong M., Li W. (2022). Structural and prebiotic activity analysis of the polysaccharide produced by *Lactobacillus helveticus* SNA12. Carbohydr. Polym..

[45] Abid Y., Casillo A., Gharsallah H., Joulak I., Lanzetta R., Corsaro M.M., Attia H., Azabou S. (2018). Production and structural characterization of exopolysaccharides from newly isolated probiotic lactic acid bacteria. Int. J. Biol. Macromol..

[46] Vazquez-Rodriguez A., Vasto-Anzaldo X.G., Barboza Perez D., Vázquez-Garza E., Chapoy-Villanueva H., García-Rivas G., Garza-Cervantes J.A., Gómez-Lugo J.J., Gomez-Loredo A.E., Garza Gonzalez M.T. (2018). Microbial Competition of *Rhodotorula mucilaginosa* UANL-001L and *E. coli* increase biosynthesis of Non-Toxic Exopolysaccharide with Applications as a Wide-Spectrum Antimicrobial. Sci. Rep..

[47] Kanmani P., Suganya K., Kumar R.S., Yuvaraj N., Pattukumar V., Paari K.A., Arul V. (2013). Synthesis and functional characterization of antibiofilm exopolysaccharide produced by *Enterococcus faecium* MC13 isolated from the gut of fish. Appl. Biochem. Biotechnol..

[48] Ying M., Yu Q., Zheng B., Wang H., Wang J., Chen S., Nie S., Xie M. (2020). Cultured *Cordyceps sinensis* polysaccharides modulate intestinal mucosal immunity and gut microbiota in cyclophosphamide-treated mice. Carbohydr. Polym..

[49] Tian J., Zhao X., Tang C., Wang X., Zhang X., Xiao L., Li W. (2023). Protective effect of *Paecilomyces cicadae* TJJ11213 exopolysaccharide on intestinal mucosa and regulation of gut microbiota in immunosuppressed mice. Food Res. Int..

[50] Kšonžeková P., Bystrický P., Vlčková S., Pätoprstý V., Pulzová L., Mudroňová D., Kubašková T., Csank T., Tkáčiková Ľ. (2016). Exopolysaccharides of *Lactobacillus reuteri*: Their influence on adherence of *E. coli* to epithelial cells and inflammatory response. Carbohydr. Polym..

[51] Pramudito T.E., Klostermann C., Smid E.J., Schols H.A. (2025). Modulation of soy flour bioactivity against enterotoxigenic *Escherichia coli* by fermentation with exopolysaccharides-producing lactic acid bacteria. Carbohydr. Polym..

[52] Nehal F., Sahnoun M., Smaoui S., Jaouadi B., Bejar S., Mohammed S. (2019). Characterization, high production and antimicrobial activity of exopolysaccharides from *Lactococcus lactis* F-mou. Microb. Pathog..

[53] Rahbar Saadat Y., Yari Khosroushahi A., Pourghassem Gargari B. (2019). A comprehensive review of anticancer, immunomodulatory and health beneficial effects of the lactic acid bacteria exopolysaccharides. Carbohydr. Polym..

[54] Bhat B., Bajaj B.K. (2019). Hypocholesterolemic potential and bioactivity spectrum of an exopolysaccharide from a probiotic isolate *Lactobacillus paracasei* M7. Bioact. Carbohydr. Diet. Fibre.

[55] Sharma V., Ghosh M. (2021). Characterization of immunomodulatory, anticancer and antioxidant properties of an extracellular polymer produced by *Enterococcus* sp. in vegetable waste medium. Environ. Sustain..

[56] Choudhuri I., Khanra K., Pariya P., Maity G.N., Mondal S., Pati B.R., Bhattacharyya N. (2020). Structural Characterization of an Exopolysaccharide Isolated from *Enterococcus faecalis*, and Study on its Antioxidant Activity, and Cytotoxicity Against HeLa Cells. Curr. Microbiol..

[57] Andrew M., Jayaraman G. (2020). Structural features of microbial exopolysaccharides in relation to their antioxidant activity. Carbohydr. Res..

[58] Ledwaba S.E., Bolick D.T., de Medeiros P., Kolling G.L., Traore A.N., Potgieter N., Nataro J.P., Guerrant R.L. (2022). Enteropathogenic *Escherichia coli* (EPEC) expressing a non-functional bundle-forming pili (BFP) also leads to increased growth failure and intestinal inflammation in C57BL/6 mice. Braz. J. Microbiol..

[59] Li X., Chen Y., Peng X., Zhu Y., Duan W., Ji R., Xiao H., Li X., Liu G., Yu Y. (2024). Anti-inflammation mechanisms of a homogeneous polysaccharide from *Phyllanthus emblica* L. on DSS induced colitis mice via the gut microbiota and metabolites alteration. Food Chem..

[60] Zhu Y., Zhou J., Liu W., Pi X., Zhou Q., Li P., Zhou T., Gu Q. (2021). Effects of exopolysaccharide from *Lactobacillus rhamnosus* on human gut microbiota in in vitro fermentation model. LWT.

[61] Peng P., Feng T., Yang X., Ding R., Wang J., Chen P., Guo Y., Li P. (2024). Bioorthogonal conjugation and responsive nanocoating of probiotics for inflammatory bowel disease. J. Control. Release.

[62] Xie A., Ji H., Liu Z., Wan Y., Zhang X., Xiong H., Nie S.P., Wan H. (2023). Modified Prebiotic-Based “Shield” Armed Probiotics with Enhanced Resistance of Gastrointestinal Stresses and Prolonged Intestinal Retention for Synergistic Alleviation of Colitis. ACS Nano.

[63] Zhou Z., Zhang M., Yao M., Naseeb J., Sarwar A., Yang Z., Aziz T., Alhomrani M., Alsanie W.F., Alamri A.S. (2024). *Lactiplantibacillus plantarum* NMGL2 exopolysaccharide ameliorates DSS-induced IBD in mice mainly by regulation of intestinal tight junction and NF-κB p65 protein expression. Front. Microbiol..

[64] Chen Y., Zhang M., Ren F. (2019). A Role of Exopolysaccharide Produced by *Streptococcus thermophilus* in the Intestinal Inflammation and Mucosal Barrier in Caco-2 Monolayer and Dextran Sulphate Sodium-Induced Experimental Murine Colitis. Molecules.

[65] Gao J., Li Q., Liu Y., Yang B., Ahmed Sadiqb F., Li X., Mi S., Sang Y. (2022). Immunoregulatory effect of *Lactobacillus paracasei* VL8 exopolysaccharide on RAW264.7 cells by NF-κB and MAPK pathways. J. Funct. Foods.

[66] Li J., Li Q., Wu Q., Gao N., Wang Z., Yang Y., Shan A. (2023). Exopolysaccharides of *Lactobacillus rhamnosus* GG ameliorate *Salmonella typhimurium*-induced intestinal inflammation via the TLR4/NF-κB/MAPK pathway. J. Anim. Sci. Biotechnol..

[67] Wang K., Niu M., Yao D., Zhao J., Wu Y., Lu B., Zheng X. (2019). Physicochemical characteristics and in vitro and in vivo antioxidant activity of a cell-bound exopolysaccharide produced by *Lactobacillus fermentum* S1. Int. J. Biol. Macromol..

[68] Huang Q., Liang J., Yang C., Li K., Niu M., Fan J., Zhang X. (2023). Stimulation-responsive mucoadhesive probiotics for inflammatory bowel disease treatment by scavenging reactive oxygen species and regulating gut microbiota. Biomaterials.

