# Supplementary material for: Gut-Protective and Multifunctional Exopolysaccharide from Enterococcus faecium HDRsEf1: Structural Characterization and Protective Effects Against Enteropathogenic E. coli-Induced Intestinal Inflammation
Source: Nutrients. 2025 Nov 24;17(23):3667. doi: 10.3390/nu17233667 (PMC12694250; doi:10.3390/nu17233667)
Supplement: Supplementary file 1 [file nutrients-17-03667-s001.zip › nutrients-3967580-supplementary.pdf]

Supplementary Materials

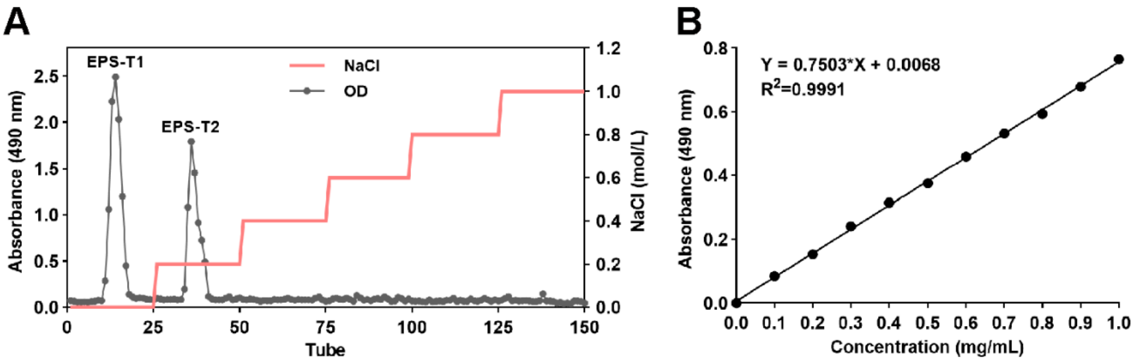

Figure S1. Characterization of EPS fractions. **(A)** Elution profiles of crude EPS on DEAE-650 M anion-exchange column; **(B)** Calibration curve for total sugar content determined by the phenol–sulfuric acid method (glucose as standard).

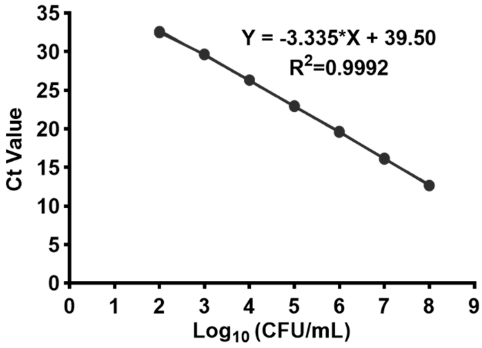

Figure S2. Standard curve constructed based on the *luxA* gene for quantification of bacterial load.

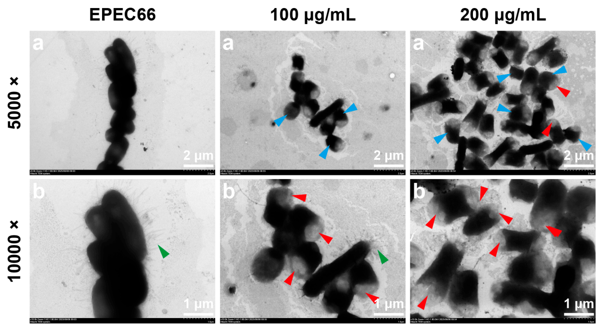

Figure S3. Negative staining TEM images of bacteria (a, 5000×; b, 10000×); uneven cytoplasmic density (red arrows), pili (green arrows), swollen or deformed cells (blue arrows).

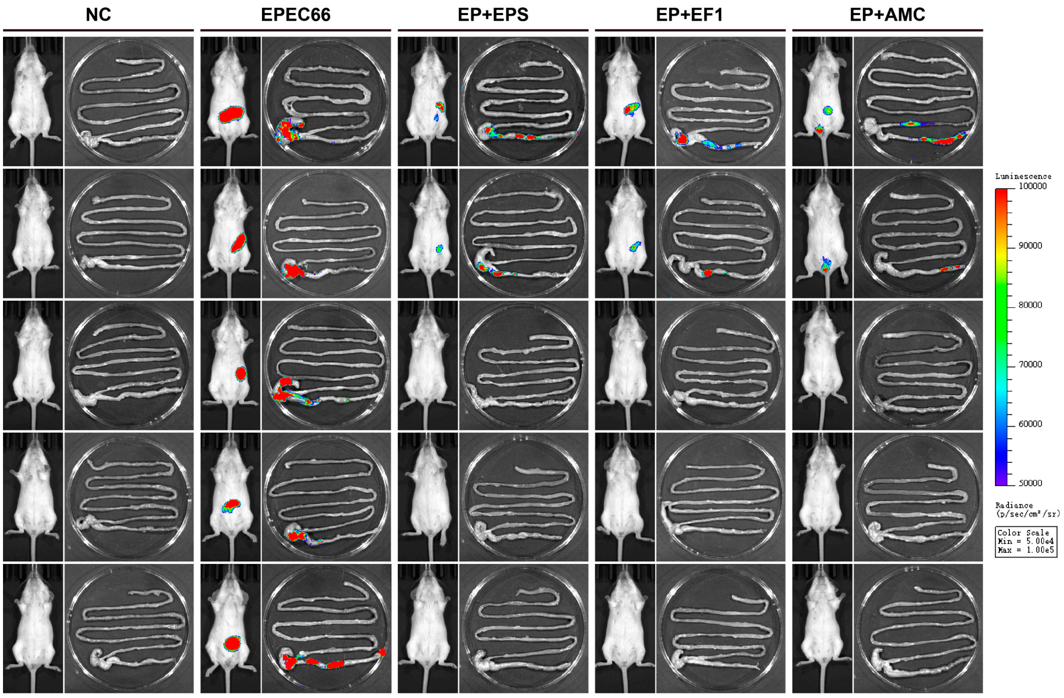

Figure S4. In vivo and intestinal bioluminescence imaging of mice infected with EPEC66-lux (n = 5), pseudocolor heat maps indicate bioluminescence intensity from low (blue) to high (red).

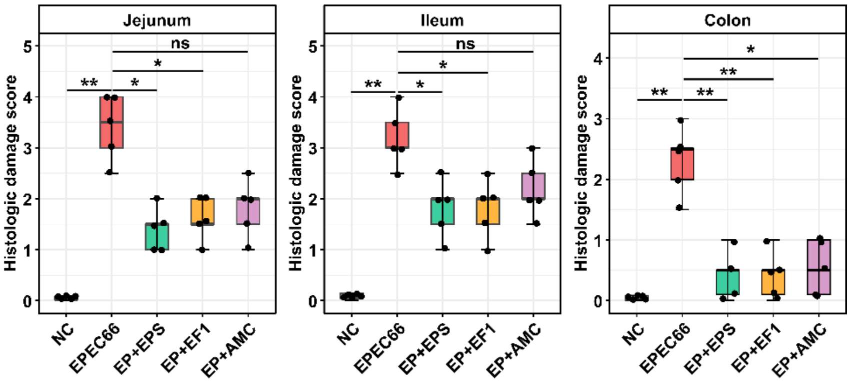

Figure S5. Histologic damage score (n = 5).

Table S1. Primers for detecting inflammation.

Gene	Primer Sequences (5'-3')	RefSeq ID
Rplp0	F: TGACATCGTCTTTAAACCCCG<br>R: TGTCTGCTCCCACAATGAAG	NM_007475.5
TNF-α	F: GGTGCCTATGTCTCAGCCTCTT<br>R: GCCATAGAACTGATGAGAGGGAG	NM_013693.3
IL-1β	F: TGGACCTTCCAGGATGAGGACA<br>R: GTTCATCTCGGAGCCTGTAGTG	NM_008361.4
IL-6	F: TACCACTTCACAAGTCGGAGGC<br>R: CTGCAAGTGCATCATCGTTGTTC	NM_031168.2
IL-10	F: CGGGAAGACAATAACTGCACCC<br>R: CGGTTAGCAGTATGTTGTCCAGC	NM_010548.2

Table S2. Molecular weight distributions

Sample	Mw (kDa)	Mn (kDa)	Đ
EPS-T1	81.21 ± 1.28	19.72 ± 0.76	4.13 ± 0.14
EPS-T2	57.79 ± 1.07	10.06 ± 0.34	5.76 ± 0.11

[1] Mw: weight-average molecular weight; Mn: number-average molecular weight; Đ: dispersity.

Table S3. Monosaccharide composition (%)

Sample	Glc	Gal	GalA	GlcA	Man	Rha
EPS-T1	61.86 ± 1.19	7.13 ± 0.18	_[a]	_[a]	24.95 ± 0.95	6.36 ± 0.11
EPS-T2	56.74 ± 1.08	_[a]	34.61 ± 0.67	8.82 ± 0.22	_[a]	_[a]

[2] _[a] Not detected. Rha: rhamnose; Man: mannose; Glc: glucose; Gal: galactose; GlcA: glucuronic acid; GalA: galacturonic acid.
